# Genotyping of Type A Human Respiratory Syncytial Virus Based on Direct F Gene Sequencing

**DOI:** 10.3390/medicina55050169

**Published:** 2019-05-20

**Authors:** Daifullah Al Aboud, Nora M. Al Aboud, Mater I. R. Al-Malky, Ahmed S. Abdel-Moneim

**Affiliations:** 1 College of Medicine, Taif University, Al-Taif 21944, Saudi Arabia; d.alaboud@tu.edu.sa; 2 Department of Biology, College of Applied Sciences, Umm Al Qura University, Makkah 21955, Saudi Arabia; nmaboud@uqu.edu.sa; 3 Hospital of Paediatrics, Al-Taif 21944, Saudi Arabia; mater1386@hotmail.com; 4 Department of Virology, Faculty of Veterinary Medicine, Beni-Suef University, Beni-Suef 62511, Egypt

**Keywords:** hRSV, F gene, G gene, children, respiratory diseases, genotyping, Saudi Arabia

## Abstract

*Background and objectives:* The human respiratory syncytial virus (hRSV) is among the important respiratory pathogens affecting children. Genotype-specific attachment (G) gene sequencing is usually used to determine the virus genotype. The reliability of the fusion (F) gene vs. G gene genotype-specific sequencing was screened. *Materials and Methods:* Archival RNA from Saudi children who tested positive for hRSV-A were used. Samples were subjected to a conventional one-step RT-PCR for both F and G genes and direct gene sequencing of the amplicons using the same primer sets. Phylogeny and mutational analysis of the obtained sequences were conducted. *Results:* The generic primer set succeeded to amplify target gene sequences. The phylogenetic tree based on partial F gene sequencing resulted in an efficient genotyping of hRSV-A strains equivalent to the partial G gene genotyping method. NA1, ON1, and GA5 genotypes were detected in the clinical samples. The latter was detected for the first time in Saudi Arabia. Different mutations in both conserved and escape-mutant domains were detected in both F and G. *Conclusion:* It was concluded that a partial F gene sequence can be used efficiently for hRSV-A genotyping.

## 1. Introduction 

Human respiratory syncytial virus (hRSV) causes considerable respiratory distress with variable severity in infancy and early childhood [1,2]. It is also responsible for the induction of respiratory illness in elderly and immunocompromised patients [3], in addition to being a common nosocomial pathogen [4]. The virus belongs to the family *Pneumoviridae,* genus *Orthopneumovirus*, and possesses a single-stranded negative-sense RNA genome that encodes eleven proteins [5], including both attachment (G) and fusion (F) envelope proteins that work together to attach to the target cell membrane by the G protein [6], while fusion of the viral and cell membranes occurs through the action of the F glycoprotein [7].

The G glycoprotein is a type II surface protein which is highly glycosylated and possesses a considerable degree of nucleotide variability [8]. It possesses two hypervariable regions (HVR1 and HVR2); the HVR2 in the C-terminal region is used to screen the G gene variability of different genotypes [9,10]. Five N-glycosylation sites were detected in HVR2 of the hRSV-A attachment protein [11]. The F protein is more conserved than the G glycoprotein and it is translated as an *F0* precursor that is cleaved twice, resulting in disulfide-linked *F1* (aa 137–574) and *F2* (aa 1–109) subunits along with a short peptide, pep27 (aa 110–136). *F2* possesses heptad repeat C (HRC), while *F1* possesses, at its N terminal, a hydrophobic fusion peptide (FP) followed by two heptad repeats, A and B (HRA and HRB). HRA, HRB, and HRC are essential for envelope fusion to the host cell membrane [12,13]. *F2* possesses five N-glycosylation sites, while HRB, part of *F1*, possesses a single N-glycosylation site; however, *F2* contains more conserved sequence than *F1,* which is characterized by a high homology among different hRSV genotypes [14]. The highly conserved nature of *F2* qualifies it for being a potential target for diagnostic assays.

The hRSV is classified into two major groups: hRSV A and hRSV B [15]. To date, 20 genotypes of hRSV-A and 36 genotypes of hRSV-B are known to exist based on the sequence variation of the G gene [16,17]. Although both hRSV types circulate worldwide, type A was found to be dominant in certain countries [18,19]. Novel genotypes may appear and could replace prevalent genotypes, and many genotypes may circulate together; however, some new genotypes may dominate [20,21]. In 1999, the B/BA genotype, with a duplication of 60 nucleotides of the G gene, appeared [22]; then, the A/ON1 genotype, with a duplication of 72 nucleotides (nt) in the G gene, appeared [23]. Both genotypes are currently prevalent worldwide. The severity of the disease is dependent on the virus genotype, as genotypes A/GA2, A/GA4, A/ON1, and B/BA were found to be of relatively low virulence in comparison to A/NA1, A/GA3, and A/GA5 [24,25,26].

Typing of hRSV is mainly based on direct G sequencing of sequences flanking HVR2; however, genotype-specific amplification is needed. The F gene could constitute an alternative method for hRSV genotyping; it was found to successfully genotype RSV strains in comparison to the G gene and resulted in the same phylogenetic clusters based on a full genome sequence of the F gene [27]. Accordingly, the aim of the current study was to screen the efficiency of genotyping hRSV-A based on gene sequencing of *F2* and the first part of *F1* (FP and HRA) and compare it with genotype-specific partial G gene sequencing. Genotyping and sequence analysis of the Saudi hRSV-A strains is another objective of the current study.

## 2. Materials and Methods

### 2.1. Ethical Statement

Ethical release was obtained from both by the College of Medicine, Taif University (TU/1652/1433/1, approved on 29th April 2011), and also from the hospital of Paediatrics ethical committee based on the human subjects protection guidelines. The first part of the study was previously published [28]. The guardians of the children involved in the study signed informed written consent about the study.

### 2.2. Samples

A total of 59 archived RNA/DNA samples that showed positive real-time RT-PCR results for hRSV-A, determined in our previous study [28], were used as targets for amplification of F and G genes. The RNA samples were stored at −80 °C. All samples were collected previously from children with respiratory distress from hospital of Paediatrics, Taif, Saudi Arabia, during the period from January to May 2012. The viral load in the samples (*n* = 56) samples were found to be weak, with a range of 1 × 10^2^ to 1 × 10^4.5^ copies/mL [29].

### 2.3. One-Step Conventional RT-PCR

An F gene-specific oligonucleotide primer set: Fus-For (1–29) 5′-ATGGAGTTGCCAATCCTCAAAGCAAATGC-3′ and Fus-Rev (622–643) 5′-ATATGCTGCAGCTTTGCTTGTT-3′, that flanks F2 and the first part of F1 (FP and HRA), was designed based on hRSV-A-GZ08-0 (KP218910). G gene genotype-specific primers for ON1/NA1 and GA5 that flank HVR2 in the C-terminal region of the G gene were also designed and used for amplification of the partial G gene based on the result of F gene sequencing. G gene-specific primer sets were as follows: ON1/NA1 (365-384)-For 5′-CTGAGTCAACCCCACAATCC-3′, ON1/NA1-Rev (24–43 G–F intergene UTR) 5′-ATTTGGTCATGGCTTTTTGC-3′ based on based on hRSV-A-GZ08-0 (KP218910) and GA5-For (374–395) 5′-TCCTGCAATCTACAACAGTCAA -3′ and GA5-Rev-(867–889) 5′-CTGTTATGTTGGATGGAGATG-GA-3′ based on RSVA/Homo sapiens/ITA/120/2009 (KF826832) were designed. Reverse transcription was conducted using GoTaq^®^ 1-Step RT-PCR (Promega, Southampton, UK) that was based on GoScript™ Reverse Transcriptase. Briefly, the program was adjusted for reverse transcription at 45 °C for 45 min, followed by an initial denaturation step at 95 °C for 5 min and 35 cycles of 95 °C for 30 s, 50 °C for 30 s, and 72 °C for 1 min. This was followed by a final 10 min elongation step at 72 °C. The RT-PCR amplicons were subjected to 1.5% agarose gel electrophoresis.

### 2.4. Direct Sequencing and Gene Sequence Analysis

Amplicons with the expected size were excised from the gel and then purified using a gel-extraction kit (Koma Biotek, Seoul, Korea). The purified amplicons were used as templates for direct sequencing using the same primers as those used for the F and G genes’ amplification. Sequencing was performed commercially using the BigDye v.3.1 Applied Biosystems (Foster City, CA, USA) kit according to the manufacturer’s protocol. Raw sequences were visualized and analyzed using MEGA 5.2. Homology BLASTn searches from different strains were performed using highly similar sequences (megablast) against published hRSV sequences in the GenBank databases using default algorithm parameters. The gene sequences of both F and G genes were deposited in GenBank under the accession numbers: F (KU924011-KU924023) and G (MK182708-MK182720). CLUSTAL W multisequence analysis was performed and the phylogenetic tree was constructed using the maximum likelihood statistical method with 1000 bootstrap replicates and Tamura–Nei model substitution model. Nucleotide variability among different strains was calculated using the CLUSTALW available in GenomeNet Database [30].

### 2.5. Deduced Amino Acid Sequence and Sequence Analysis

Deduced amino acid sequences of both F and G amino acid sequences were compared using MEGA 5.2. Amino acid substitutions at different functional and structural protein fragments were analysed. The polyphen (polymorphism phenotyping) prediction webtool [31] was used to screen the potential effect of amino acid substitutions among strains on the function.

## 3. Results

### 3.1. Direct Sequencing and Phylogenetic Analysis

**Figure 1:**
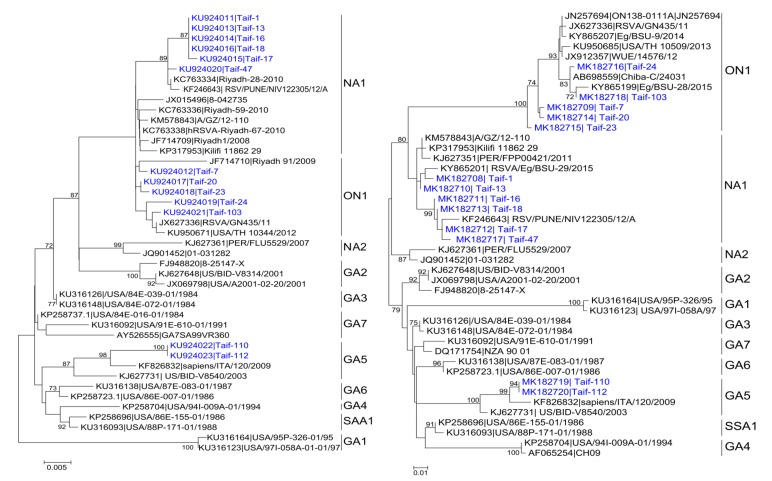
Phylogenetic trees of the nucleotide sequences of partial fusion (F) and attachment (G) genes of human respiratory syncytial virus A (hRSV-A) strains from Saudi Arabia in comparison to relevant published strains. (**a**) F gene; (**b**) G gene. The maximum likelihood tree was constructed using MEGA 5.2 freeware. Strains detected in the current study are shown in blue color. Only bootstrap values above 70% are shown.

Both generic primers for F and G genes were successful in amplification of the strains, showing relatively high viral load for 13 of the 59 samples (≥1 × 10^3.5^ copies/mL). The F gene domains of the hRSV-A isolates possess about 10% nucleotide variability. The signal peptide, however, showed higher nucleotide variability. Partial F gene sequencing revealed that the Saudi strains in the current study belong to the NA1 genotype (6/13, 46.15%), ON1 (5/13; 38.46%), and GA5 genotype (2/13; 15.38%) (Figure 1a). Partial G gene sequencing using strain-specific primers for these strains confirmed the correct strain classification obtained by using F gene sequencing, and similar clustering of the hRSV strains was obtained using both genes (Figure 1a,b). The F gene domains of the hRSV-A isolates possess about 10% nucleotide variability. The signal peptide and transmembrane domains, however, showed nucleotide variability to be higher than detected in the HVRs of the G gene (data not shown).

### 3.2. Deduced Amino Acid Sequence and Sequence Analysis

**Figure 2:**
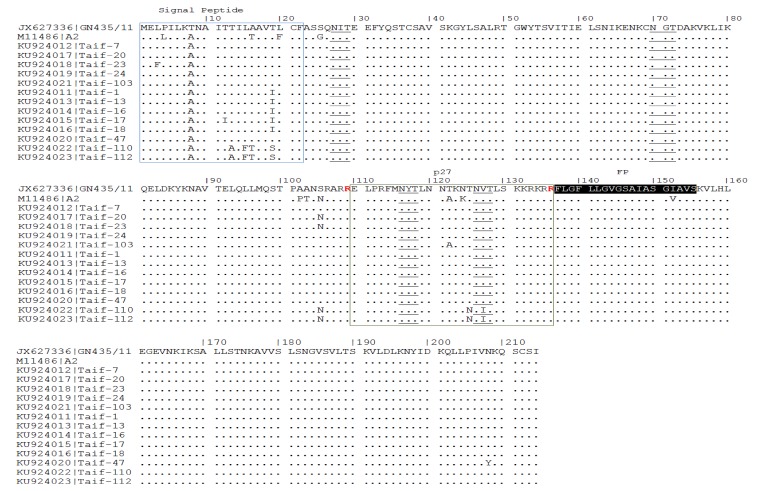
Deduced amino acid sequences of partial fusion proteins from different Saudi hRSV-A strains. Highlighted are the signal peptide (1–22) (blue box), heptad repeat domain C (75–97), cleavage site-1 (CS-1) and cleavage site-2 (CS-2) at Arg109 (NSRARR↓E) and Arg136 (KKRKRR↓F) (red color), p27 (110–136) (green box), fusion peptide (FP) (137–155), and heptad repeat domain A (153–end of the current sequence). The N-glycosylation sites, NXT/S, where X is not a proline, are underlined.

Five N-glycosylation sites, N27, N70, N116, N120, and N126, were found to be conserved among all the thirteen Saudi strains, except for strain Taif-103, that has lost one of the N-glycosylations at N120 (Figure 2). The fusion protein of Taif-20, Taif-23, Taif-110, and Taif-112 showed S-to-N substitution at position 105 just before the first cleavage (Figure 2), with no potential effect on the function as demonstrated by the polymorphism phenotyping prediction tool (data not shown). Seven amino acid substitutions—L3F, T11I, I19T, T13A, L15F, A16T, and I19T—were detected in the signal peptide, and three—T120A, T125N, and V127I—amino acid substitutions were detected in the p27 domain (Figure 2).

**Figure 3:**
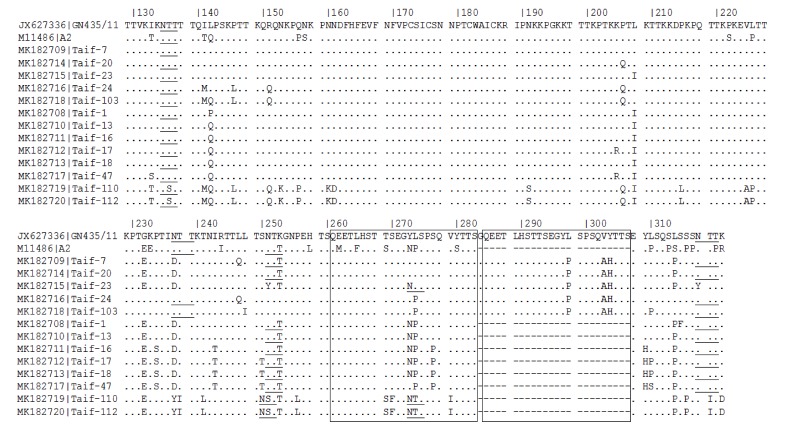
Deduced amino acid sequences of the partial attachment protein (G) from different Saudi hRSV-A strains. The N-glycosylation sites, NXT/S, where X is not a proline, are underlined. Duplicated regions in the ON1 strains are shown in the boxes.

A duplication of amino acids (QEETLHSTTSEGYLSPSQVYTTS) was found in two regions within the G protein which reflects an 72 nt insert characteristic to the ON1 genotype (Figure 3). L298P, V303A, and Y304H amino acid substitutions were detected in all ON1 Saudi strains, Y273N was detected in Taif 23, and L274P was detected in Taif-24 and Taif-103 strains (Figure 3). The P310L substitution, equivalent to P286L in viruses that have no duplication, was recorded in 10 out of the 13 Saudi strains. There is an existence of accumulated signature coding changes in the epitope regions of the G protein (Figure 3). Variations among the N-glycosylation site of HVR2 of the Saudi strains’ attachment proteins were detected. Interestingly, all Saudi NA1 and ON1 strains except Taif-24 and Taif-103 showed N237D amino acid substitution that resulted in loss of the N237 N-glycosylation site. N250 was found only in Saudi GA5 strains, while N251 was detected in 2/5 ON1 and 6/6 of the NA1 Saudi strains. N273 was detected only in Saudi GA5 strains, as well as a single ON1 Saudi strain. N294 (N318 in ON1 genotypes) was detected in the majority of Saudi ON1 and NA1 strains, but none of the Saudi GA5 strains or the A2 prototype strain (Figure 3).

## 4. Discussion

Both of the designed generic primers were successful in amplification of the three different genotypes of hRSV-A, ON1, NA1, and GA5, that were detected among Saudi archival RNA samples. It is also assumed that the generic F primer set could detect all hRSV-A strains, since it showed complete identities to most of the published hRSV-A strains except for a single mismatch detected occasionally. Genotyping based on partial gene sequencing of both F and G genes showed similar results, which denotes that the designed F gene could be used efficiently in genotyping of hRSV-A strains.

The Saudi strains in the current study are classified as NA1, ON1, and GA5 genotypes. Both NA1 and GA5 showed higher virulence to the children in comparison to ON1 and other hRSV-A strains [24,25,26]. Two out of the thirteen strains were related to GA5, and this represents the first record of this subtype in Saudi Arabia. In Saudi Arabia, only a few studies have been conducted on hRSV genotypes; however, the hRSV-A type, mostly the NA-1 subtype, is the dominant genotype [32,33]. The hRSV-A ON1 genotype was detected for the first time in Canada in 2010 [23]. A Saudi strain in the GenBank database was found to be closely related to the ON1 strain isolated in 2009 in Riyadh and also closely related to ON1 strains detected in the current study from Al-Taif. This finding reveals that the ON1 strains have circulated in Saudi Arabia since 2009. This assumption is confirmed by the global data that suggested that the ancestor of ON1 emerged during 2008–2009 [34].

It is known that the F protein is focused on the signal peptide, p27, transmembrane domain, and ø antigenic site [35]. Similarly, the highest variability among amino acids among Saudi strains was detected in the signal peptide, followed by the p27 domain of the fusion protein. The fusion protein of hRSV strain harbors six N-glycosylation sites [9,36], which were confirmed to be conserved among the current Saudi strains, except for strain Taif-103 (A/ON1), which has lost one of the N-glycosylations at N120. It is known that N-glycosylation is important for the folding and transport of viral proteins and hence virus infectivity [37]. It is worth mentioning here that the three N-glycosylation sites—N116, N120, and N126—are found in close proximity to the proteolytic cleavage site [38,39]. It seems that the T122A amino acid substitution that resulted in a loss of the N-glycosylation site in Taif-103 does not affect the fusion or the proteolytic affinity of the protein, as confirmed previously [38].

The deduced amino acid sequence of the G gene revealed the presence of L298P, V303A, and Y304H amino acid substitution in all ON1 Saudi strains. Y273N was detected in Taif 23 and L274P was detected in the Taif-24 and Taif-103 strains. Such substitutions are considered noteworthy as they are present next to aa 265–273 (antigenic site) [40]. The P310L was recorded in the majority of the Saudi strains. Positively selected residues reported by Botosso et al., were found to be conserved in the Saudi ON1 and NA1 strains, except at the residues T237D and L274P [41] that constituted escape-mutant screened monoclonal antibodies [42,43]. The N273 N-glycosylation site was lost in the Saudi NA1 and most of the ON1 strains, which was also observed in NA1 strains from Japan and China [11,20]. Absence of the N250 site in all Saudi strains except for Saudi GA5 strains was expected, since it is a specific N-glycosylation site for both GA5 and SAA1 of hRSV-A [11]. The variation of the number of N-glycosylation sites was found to be associated with altered antigenicity [44].

## 5. Conclusions

Direct partial F gene sequencing represents an accurate method for hRSV-A genotyping that matched partial G gene sequencing results. The current study provides evidence of the circulation of GA5, NA1, and ON1 genotypes in Saudi Arabia. Although mutations in conserved or escape-mutant domains were detected in both F and G proteins, most of them do not affect the virus’ virulence.
